# Characterization of HIV-1 uncoating in human microglial cell lines

**DOI:** 10.1186/s12985-020-01301-5

**Published:** 2020-03-06

**Authors:** Zachary Ingram, Melanie Taylor, Glister Okland, Richard Martin, Amy E. Hulme

**Affiliations:** Department of Biomedical Sciences, Missouri State University, Springfield, MO USA

**Keywords:** Human immunodeficiency virus, HIV-1, Uncoating, Capsid, CA, Microglia

## Abstract

**Background:**

After viral fusion with the cell membrane, the conical capsid of HIV-1 disassembles by a process called uncoating. Previously we have utilized the CsA washout assay, in which TRIM-CypA mediated restriction of viral replication is used to detect the state of the viral capsid, to study the kinetics of HIV-1 uncoating in owl monkey kidney (OMK) and HeLa cells. Here we have extended this analysis to the human microglial cell lines CHME3 and C20 to characterize uncoating in a cell type that is a natural target of HIV infection.

**Methods:**

The CsA washout was used to characterize uncoating of wildtype and capsid mutant viruses in CHME3 and C20 cells. Viral fusion assays and nevirapine addition assays were performed to relate the kinetics of viral fusion and reverse transcription to uncoating.

**Results:**

We found that uncoating initiated within the first hour after viral fusion and was facilitated by reverse transcription in CHME3 and C20 cells. The capsid mutation A92E did not significantly alter uncoating kinetics. Viruses with capsid mutations N74D and E45A decreased the rate of uncoating in CHME3 cells, but did not alter reverse transcription. Interestingly, the second site suppressor capsid mutation R132T was able to rescue the uncoating kinetics of the E45A mutation, despite having a hyperstable capsid.

**Conclusions:**

These results are most similar to previously observed characteristics of uncoating in HeLa cells and support the model in which uncoating is initiated by early steps of reverse transcription in the cytoplasm. A comparison of the uncoating kinetics of CA mutant viruses in OMK and CHME3 cells reveals the importance of cellular factors in the process of uncoating. The E45A/R132T mutant virus specifically suggests that disrupted interactions with cellular factors, rather than capsid stability, is responsible for the delayed uncoating kinetics seen in E45A mutant virus. Future studies aimed at identifying these factors will be important for understanding the process of uncoating and the development of interventions to disrupt this process.

## Background

After fusion of the viral membrane the conical capsid of HIV is released into the cytoplasm of the cell. This capsid contains approximately 1500 monomers of the viral capsid protein (CA) arranged in a hexameric lattice around the viral RNAs and associated proteins [1–3]. In order for infection to progress this conical capsid structure disassembles by a process called uncoating. During this time, the viral RNA is reverse transcribed into a double stranded DNA. The viral complex of nucleic acid and associated proteins also must traffic through the cytoplasm on microtubules [4–6]. This viral complex then gains access to the nucleus through a nuclear pore and the viral DNA is integrated into the chromosomal DNA to establish infection of a cell. Recent evidence suggests that CA and uncoating are at the crossroads of a complex interplay between these early steps of HIV replication. Some CA mutations can disrupt reverse transcription, but blocking reverse transcription also delays uncoating [7–10]. Disruption of protein interactions required for cytoplasmic trafficking affects the extent of uncoating [11–19]. Finally, determinants for nuclear import and integration site selection map to the CA protein [20–23], but the viral capsid is too large to enter the nuclear pore. Some amount of CA protein is proposed to associate with the viral complex after uncoating to facilitate integration, which has been detected in nuclear viral complexes [24–26]. Therefore, it is necessary to characterize the process of uncoating in order to fully understand the early events of HIV replication.

When, where, and how uncoating occurs is an area of active investigation and source of contention in the field. Currently there are two models for uncoating [27]. In the cytoplasmic uncoating model, uncoating occurs in the cytoplasm as the viral complex is trafficked toward the nucleus [4, 8, 28–30]. According to the nuclear pore model, uncoating occurs at the nuclear pore after docking of the intact capsid [31–34]. Distinguishing between these two models has been challenging due to the overlapping nature of the early steps of HIV replication, differing methods used to assay uncoating, and the characteristics of defective or noninfectious virions possibly confounding results. Data from studies supporting both models suggest that a pore opens to destabilize the capsid integrity and then the hexameric CA lattice disassembles, but the time lag between these events is not clear [30, 34–38]. These models are also not necessarily mutually exclusive. A recent hypothesis that draws on both models proposes that the HIV capsid is destabilized in the cytoplasm, followed by additional loss of capsid at the nuclear pore [12, 39].

The primary viral factors involved with uncoating are the CA protein and the process of reverse transcription. Mutations in CA can decrease or increase the rate of uncoating which also decreases HIV infectivity, indicating that correct timing of uncoating is required for optimal HIV replication [7, 40–44]. Reverse transcription is also necessary for uncoating as inhibiting this process delays uncoating in cultured cell based, biochemical, and microscopy based uncoating assays [8–10, 30, 37, 38, 45]. Specifically, the generation of minus strand strong stop DNA, an early product of reverse transcription, is proposed to initiate uncoating [30, 45]. Multiple cellular proteins have been found to play at least an indirect role in uncoating [11, 12, 27]. Disruption of the interaction between eukaryotic translation elongation factor 1A (eEF1A) and reverse transcriptase delays uncoating, further highlighting the interplay between reverse transcription and uncoating [46]. Knockdown of proteins involved with microtubule trafficking can delay uncoating and impair cytoplasmic trafficking of viral complexes, including the motor proteins dynein and kinesin-1 Kif5B, kinesin-1 adaptor protein FEZ1, microtubule affinity-regulating kinase 2 (MARK2), dynein adaptor protein BICD2, and cytoskeletal regulatory proteins Dia1 and Dia2 [13–19]. In addition, knockdown of the nuclear pore protein NUP358 disrupts cytoplasmic trafficking, nuclear import, and uncoating [13, 19]. The proteins BICD2, Dia1, Dia2, FEZ1, MARK2, and NUP358 also have been shown to directly bind to the capsid to mediate these effects, indicating a direct effect on uncoating [13–15, 18, 19]. Collectively these results suggest that uncoating must occur within the correct spatiotemporal context so that the viral complex can interact with cellular factors for productive HIV infection.

We have previously examined the kinetics of uncoating in owl monkey kidney (OMK) cells and HeLa cells using the CsA washout assay [8, 40]. This assay utilizes the activity of the restriction factor TRIM-CypA to bind to the HIV capsid and then inhibit infectivity [8, 47]. Withdrawal of the drug cyclosporine A (CsA) is used to activate this TRIM-CypA restriction at various times post-infection, allowing the kinetics of uncoating to be revealed. The CsA washout assay is indirect in that relies on the activity of TRIM-CypA to detect uncoating rather than directly measuring the level of CA protein. However, fluorescence microscopy based uncoating assays and biochemical uncoating assays that directly detect the loss of CA have confirmed a similar timing, effect of reverse transcription, effect of CA mutations, and effect of cellular factors on uncoating [8–10, 16, 30, 38, 45, 48]. Therefore, while indirect the CsA washout assay provides a good monitor for the kinetics of successful uncoating in productively infected cells. An additional strength of the CsA washout assay is that it provides a direct correlation between uncoating and infectivity. In order for an uncoating event to be detected, the virus must successfully uncoat and integrate its DNA into the host cell DNA to establish productive infection. The majority of HIV virions that enter cells do not establish productive infection. The characteristics of these defective virions could bias or obscure results in uncoating studies where a large number of virions are surveyed without accounting for productive infection [29, 36–38, 48–51].

We previously used the CsA washout assay to examine the effect of different CA mutations on uncoating in infected cells [40]. These CA mutations can affect the infection of nondividing cells, utilization of nuclear import pathways, integration site selection, and interaction with cellular proteins that facilitate HIV infection [13, 20–23, 48, 52, 53]. We found that the E45A and N74D mutations uncoated slower than wildtype virus, while the mutation A92E uncoated faster than wildtype in OMK cells [40]. We also observed differential uncoating kinetics for the mutant N74D in a HeLa cell line engineered to express TRIM-CypA [40]. These results suggest that cell type differences can impact uncoating kinetics.

Given the possible role of cellular environment, here we have extended our analysis of uncoating to the human microglial cell lines CHME3 and C20 [54, 55]. Microglial cells are natural targets of HIV infection in humans and serve as a major viral reservoir in the central nervous system [56]. Infection and activation of microglial cells is proposed to be responsible for AIDS associated dementia and other neurocognitive defects observed in some AIDS patients [56]. There has also been recent interest to use cultured microglial cell models to study HIV latency [55]. Therefore, it is important to characterize HIV uncoating in this natural target of HIV infection. Given our previous experiments in OMK and HeLa cells, we examined the kinetics of uncoating, role of reverse transcription, and the effect of different CA mutants on the process of uncoating in these cultured microglial cell lines.

## Material and methods

### Cell lines, viruses, and pharmaceuticals

293 T cells were obtained from the American Type Culture Collection and were cultured in Dulbecco’s Modified Eagle Media (Cellgro), 10% fetal bovine serum (Atlanta Biologicals), and 1% Pen/Strep/Glutamine (Gibco). CHME3 cell line was a gift M. Naghavi (Northwestern University [54, 57]). The C20 cell line was a gift from D. Alvarez-Carbonell (Case Western Reserve University [55]). Prior to this study these cell lines were previously established from human microglia [54, 55]. The stable cell lines CHME3-TC and C20-TC were made using an HA-tagged TRIM-CypA expressing retroviral vector (pLXSN-TRIMCypA) to infect CHME3 and C20 cells [58]. Neomycin resistant clonal cell lines (CHME3-TC) or hygromycin resistant clonal cell lines (C20-TC) were screened for TRIM-CypA expression by western blot against HA and restriction activity. CHME3, CHME3-TC, C20 and C20-TC cells were cultured in Dulbecco’s Modified Eagle Media (Cellgro), 5% fetal bovine serum (Atlanta Biologicals), 1% Pen/Strep/Glutamine (Gibco), and 1% sodium pyruvate (Gibco). All cell lines were maintained at 37 °C and 5% CO_2_. VSV-g pseudotyped GFP reporter virus was produced by PEI transfection of 293 T cells with 6 μg HIV-1 proviral plasmid HIV-GFP or proviral CA mutant plasmid and 4 μg VSV-g expression plasmid. Virus was harvested 48 h post-transfection, purified through a 0.45 μm filter, and stored at − 80 °C until use. Cyclosporine A (Calbiochem) was prepared in ethanol and used at a final concentration of 2.5 μM. Nevirapine (NIH AIDS Research and Reference Reagent Program) was prepared in DMSO and used at a final concentration of 5 μM. Ammonium chloride was prepared in double-distilled water (ddH_2_O) and used at a final concentration of 10 μM.

### CsA washout assay

The CsA washout assay was conducted as previously described [8, 59]. Briefly, CHME3-TC or C20-TC cells were plated in 96 well dishes at a density of 6000 cells /well, with each experimental or control reaction performed on triplicate wells. These cells were spinoculated with VSV-g pseudotyped HIV-GFP or CA mutant virus in the presence of CsA and 10 μg/ml polybrene for 1 h at 16 °C. After spinoculation, the inoculation media was exchanged for warm media, and CsA was washed out of the zero time point reaction by media exchange. CsA washout by media exchange continued at various times post-infection. The negative control for each time point was ethanol washout by media exchange. To examine the effect of reverse transcription on uncoating nevirapine was included in the inoculation media and washout media for the first 2 h of the experiment. After 2 h it was removed by media exchange on all reactions. Two days post-infection cells were harvested with 100 μl trypsin and fixed by the addition of 100 μl fix (4:1, 1X PBS:10% paraformaldehyde). The percentage of GFP positive cells was determined by flow cytometry using the Accuri C6 flow cytometer, averaged for each triplicate reaction, and standard error was calculated. The percentage of infected cells in the ethanol control was subtracted from the CsA reaction at each washout time point to yield the percentage of infected cells over background. The data was normalized by setting the highest percentage of GFP positive cells to 100% for each virus or condition tested. The half-life of uncoating was determined by a best fit line through the two data points flanking 50% uncoating and times were averaged from multiple independent experiments. For statistical analysis of the half-life of uncoating, the average half-life of uncoating was compared between the CA mutant virus and its corresponding wildtype CA control using an unpaired T-test.

### Viral fusion assay

CHME3-TC and C20-TC cells were plated and spinoculated with HIV-GFP virus as described in the CsA washout assay. When inoculation media was exchanged for warm media, media containing CsA and the fusion inhibitor ammonium chloride was added to the zero time point reaction. Ammonium chloride addition continued by media exchange at various times post-infection. Controls included no treatment and continuous treatment with ammonium chloride. Two days post-infection, cells were harvested and the percentage of GFP positive cells was determined as in the CsA washout assay. The half-life of fusion was determined using a best fit line between the adjacent data points and times were averaged from five independent experiments. These assay were conducted in parallel with a CsA washout assay to directly compare the half-lives of viral fusion and uncoating.

### Nevirapine addition assay

CHME3-TC cells were plated and spinoculated with HIV-GFP virus as described in the CsA washout assay. When inoculation media was exchanged for warm media, media containing CsA and the reverse transcriptase inhibitor nevirapine (NVP) was added to the zero time point reaction. NVP addition continued by media exchange at various times post-infection. Controls included continuous treatment with NVP and DMSO carrier control (no NVP treatment). Two days post-infection, cells were harvested and the percentage of GFP positive cells was determined as in the CsA washout assay. For each virus, infectivity at each timepoint was normalized by setting the percentage of infected cells in the no nevirapine control to 100%. Data from three independent experiments was averaged for each virus. For statistical analysis of the progression of reverse transcription, an unpaired T-test was used to compare the percentage of GFP positive cells between each CA mutant virus and the corresponding wildtype CA control at each timepoint tested.

### CsA addition assay

The parent CHME3 cell line used to generate CHME3-TC cells was plated as described in the CsA washout assay. Cells were spinoculated with VSV-g pseudotyped HIV-GFP in the presence of 10 μg/ml polybrene for 1 h at 16 °C. When this inoculation media was exchanged for warm media, media containing CsA or the carrier control ethanol was added to the zero time point reaction. CsA or ethanol addition continued by media exchange at various times post-infection. Controls included no treatment and continuous treatment for both the CsA and ethanol conditions. Cells were harvested and the percentage of GFP positive cells was determined as in the CsA washout assay. The ratio of infectivity was calculated by dividing the percentage of GFP positive cells in the CsA reaction by the percentage of GFP positive cells in the ethanol control reaction for each time point.

## Results

### Kinetics of uncoating in microglial cells

To examine the kinetics of uncoating in microglial cells, we performed the CsA washout assay [8, 47]. In this assay the HIV restriction factor TRIM-CypA is used to detect uncoating [60, 61]. To restrict infection multiple TRIM-CypA proteins assemble in a lattice to bind the hexameric array of CA protein found in the assembled HIV-1 capsid [60, 62–64]. Therefore, TRIM-CypA will bind and inhibit the infectivity of coated viral complexes. The drug cyclosporine A (CsA) prevents the binding of TRIM-CypA to the viral capsid in a reversible fashion [47, 60, 61, 65]. In the CsA washout assay, cells are synchronously infected with a GFP reporter virus in the presence of CsA. When CsA is then removed at various times post-infection, TRIM-CypA can bind to any coated viral complexes and inhibit infectivity. However, viral complexes that have progressed through uncoating such that they are resistant to TRIM-CypA binding and restriction will still be able to infect the cell. Two days post-infection cells are harvested and flow cytometry is used to determine the percentage of infected cells at each time point. As only uncoated viral complexes can establish productive infection during TRIM-CypA restriction, this percentage of infected cells correlates to the percentage of uncoated virions at each timepoint [8].

**Fig. 1:**
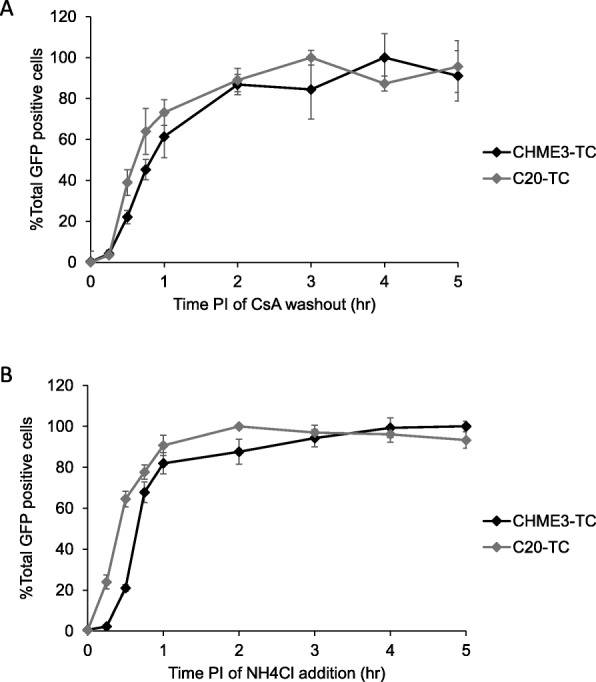
Kinetics of uncoating and viral fusion in CHME3-TC and C20-TC cells. CsA washout assays and viral fusion assays were performed in parallel to correlate the kinetics of uncoating and viral fusion in CHME3-TC and C20-TC cells. Shown is a representative experiment from five independent parallel experiments. Errors bars denote standard error among triplicate wells. **a** The uncoating kinetics were similar in both cell lines with the majority of virus uncoating within 2 h post-infection. **b** The kinetics of viral fusion were examined by the addition of ammonium chloride at various times post-infection to block viral fusion. In both cell lines the majority of virus fused within 1 h post-infection

We engineered the human microglial cell lines CHME3 and C20 to express TRIM-CypA so that the CsA washout assay could be used to study uncoating [54, 55]. The CHME3 cell line, also present in the literature as HMC3, has been verified by a recent study and the ATCC as a human microglial cell line [57]. The C20 cell line has been more recently established from adult human cortical microglia and has been characterized as human microglial by morphology, surface marker expression, RNA expression profile, and sequencing [55]. CHME3 and C20 cells were infected with a retroviral vector encoding an HA-tagged version of owl monkey TRIM-CypA. A clonal stable cell line (CHME3-TC or C20-TC) was established which expressed TRIM-CypA protein at a sufficient level to restrict HIV replication. Treatment with the drug cyclosporine A (CsA) was able to relieve TRIM-CypA restriction in the CHME3-TC and C20-TC cell lines. To characterize uncoating the CsA washout assay was performed in CHME3-TC cells and C20-TC cells using the VSV-g pseudotyped GFP reporter virus HIV-GFP. In these assays, there was an increase in the percentage of uncoated viral complexes over time, which leveled off at 4–5 h post infection (Fig. 1a). In both cell lines, the majority of uncoating (~ 80%) occurred within the first 2 h of the experiment. The data were then normalized by setting the percentage of GFP positive cells where the curve levels off at 4 or 5 h to 100%, allowing a half-life of uncoating or time of 50% uncoating to be calculated (Fig. 1a). The average half-life of uncoating was 52.46 min in CHME3-TC cells and 34.95 min in C20-TC cells (Table 1).

**Table 1:** Half-lives of viral fusion and uncoating in CHME3-TC and C20-TC cells

Cell line	Fusion (min)	Uncoating (min)	Normalized uncoating (min)
CHME3-TC	43.84 (SE = 8.02)	52.46 (SE = 8.05)	8.62
C20-TC	26.30 (SE = 1.85)	34.95 (SE = 5.62)	8.65

The average half-lives of viral fusion and uncoating were determined from 5 independent parallel experiments. SE denotes standard error

We have previously shown that alterations in the timing of viral fusion can impact the perceived rate of uncoating in the CsA washout assay [8, 40]. Viral fusion immediately precedes uncoating and the time for both viral fusion and uncoating is included in the half-life of uncoating as calculated above. Therefore, differences in the rate of viral fusion between CHME3-TC and C20-TC cells could appear as different uncoating rates. To directly compare the kinetics of viral fusion and uncoating in both cell lines, we performed a series of viral fusion assays in parallel with CsA washout assays. For the viral fusion assay cells were spinoculated with HIV-GFP in the presence of CsA and then ammonium chloride was added to block VSV-g mediated viral fusion at time points corresponding to those in CsA washout assay (Fig. 1b). The average half-life of viral fusion was 43.84 min in CHME3-TC cells and 26.3 min in C20-TC cells (Table 1). After subtracting the half-life of viral fusion from the half-life of uncoating, both microglial cell lines displayed a similar normalized rate of uncoating of 8.6 min (Table 1).

### Effect of reverse transcription on uncoating kinetics

**Fig. 2:**
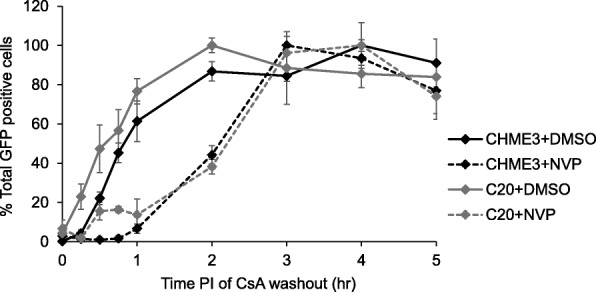
Effect of reverse transcription on uncoating in CHME3-TC and C20-TC cells. The effect of reverse transcription on the process of uncoating in CHME3-TC and C20-TC cells was determined using a 2 h treatment of nevirapine (NVP) in the CsA washout assay. Treatment with NVP delayed the process of uncoating and increased the average half-life of uncoating to 137.9 min in CHME3-TC cells and 136.4 min in C20-TC cells. Shown is a representative experiment from four independent experiments. Errors bars denote standard error among triplicate wells

To determine the effect of reverse transcription on uncoating kinetics in microglial cells, we performed the CsA washout assay using the non-nucleoside reverse transcriptase inhibitor nevirapine to block reverse transcription for the first 2 h of the assay. This 2 h nevirapine treatment delayed the process of uncoating in CHME3-TC and C20-TC cells as evidenced by a shift in the uncoating curve to the right compared to no treatment (Fig. 2). Inhibition of reverse transcription increased in the half-life of uncoating to 137.9 min in CHME3-TC cells and 136.4 min in C20-TC cells. In addition, there was a rapid increase in the percentage of uncoated viral complexes in the first hour after nevirapine removal, between the 2 to 3 h timepoints, in both cell lines (Fig. 2).

### Effect of CA mutations on uncoating

**Fig. 3:**
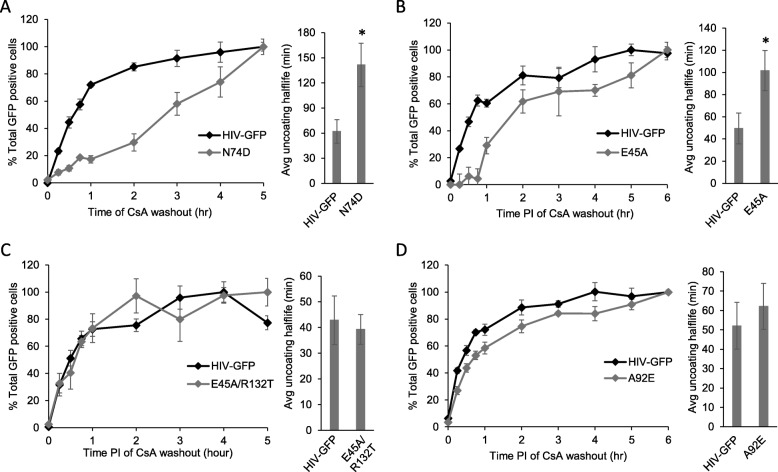
Effect of CA mutations on uncoating in CHME3-TC cells. The CsA washout assay was performed with CA mutant viruses using HIV-GFP as the wildtype CA control. Line graphs shown the uncoating kinetics from a representative CsA washout assay. Errors bars denote standard error among triplicate wells. Bar graphs compare the average half-life of uncoating of CA mutant viruses and the wildtype HIV-GFP control from multiple independent experiments. Error bars denote standard error among these independent experiments. * indicates a significant difference of *P* < 0.05. **a** The N74D mutation significantly decreased the rate of uncoating among six independent experiments. **b** The E45A mutation significantly decreased the rate of uncoating among six independent experiments. **c** The compensatory mutation R132T was able to rescue the uncoating kinetics of the E45A mutation to wildtype levels in five independent experiments. **d** The A92E mutation did not significantly alter the rate of uncoating among seven independent experiments

We previously examined the uncoating of a panel of CA mutant viruses in OMK cells and found that the N74D, A92E, and E45A mutations altered the rate of uncoating compared to wildtype [40]. These mutants are of interest due to their involvement with viral cytoplasmic trafficking, nuclear import, integration, and interaction with cellular factors [13, 20–23, 48, 52, 53]. Therefore, we examined the effect of these mutations on uncoating in microglial cells. Using the CsA washout assay, the uncoating kinetics of each CA mutant was examined in parallel to the wildtype control HIV-GFP in CHME3-TC cells. All three mutants decreased the infectivity of HIV. By normalizing the data for each mutant independently, this decreased level of infectivity does not bias uncoating kinetics in the CsA washout assay. Compared to HIV-GFP, the N74D mutation delayed the process of uncoating and increased the half-life of uncoating to 141 min compared to 62 min for wildtype (Fig. 3a). The E45A mutation also delayed the process of uncoating compared to wildtype, with an average half-life of 102 min compared to 50 min for wildtype (Fig. 3b). We also examined the uncoating of virus containing the R132T second site suppressor mutation which partially restores the infectivity of E45A mutant virus [66]. This E45A/R123T mutant virus uncoated with kinetics like wildtype, with an average half-life of uncoating of 39 min that was not statistically different from the average half-life of 43 min for wildtype in parallel experiments (Fig. 3c). Finally, A92E mutant virus had an uncoating half-life of 62 min that was not statistically different from the 52 min for wildtype (Fig. 3d).

### Effect of CA mutations on reverse transcription

**Fig. 4:**
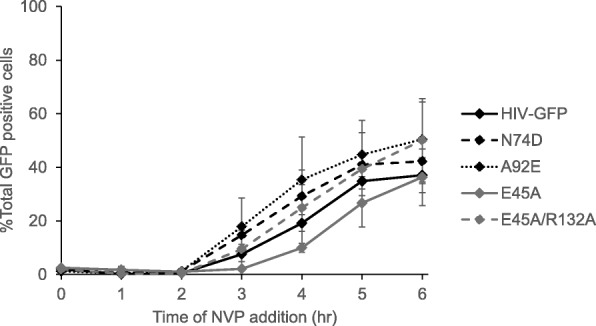
Reverse transcription kinetics of CA mutant viruses in CHME3-TC cells. Completion of reverse transcription was examined using a nevirapine addition assay. For each virus, infectivity at each timepoint was normalized to the DMSO carrier control. A statistically significant difference in reverse transcription compared to the HIV-GFP control was not found at any timepoint. Shown is the average of three independent experiments. Error bars denote standard error among these independent experiments

Blocking reverse transcription delays uncoating in CHME3-TC cells and some CA mutations can disrupt reverse transcription (Fig. 2 [7]). Therefore, changes in the uncoating kinetics of the mutant viruses could be due to alterations in reverse transcription (Fig. 3). To examine the kinetics of reverse transcription for each mutant virus, we performed an addition assay with the non-nucleoside reverse transcriptase inhibitor nevirapine (NVP; Fig. 4). In this assay, cells were spinoculated with wildtype or CA mutant GFP reporter virus in the presence of CsA and then NVP was added at time points corresponding to those in CsA washout assay. At each timepoint, virus that had completed reverse transcription would be resistant to nevirapine and able to infect the cell. The data were normalized by setting the percentage of GFP positive cells in the DMSO carrier control to 100%. Some alterations in completion of reverse transcription were observed, with A92E seeming to reverse transcribe at the greatest rate and E45A at the slowest rate (Fig. 4). However, none of these differences were found to be statistically significant compared to the HIV-GFP wildtype control at any timepoint. Therefore, these data suggest that these CA mutations do not affect completion of reverse transcription at early time points post-infection in CHME3 cells.

### Effect of CsA in CHME3 cells

**Fig. 5:**
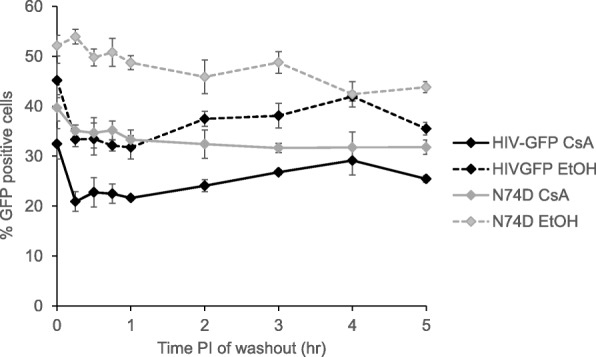
Effect of CsA on infectivity in CHME3 cells. The effect of CsA on HIV-GFP and N74D infectivity in the parent CHME3 cell line was determined by comparing CsA washout and EtOH washout at times corresponding to the CsA washout assay. The presence of CsA decreases the infectivity of both HIV-GFP and N74D virus at all time points tested. Shown is a representative assay from three independent experiments. Error bars denote standard error among triplicate wells

Cyclosporine A is used in the CsA washout assay to control TRIM-CypA mediated restriction of infection. In HeLa cells CsA treatment has been shown to decrease the infectivity of N74D mutant virus, but not virus with a wildtype capsid [67]. In the CsA washout assay, the data is normalized for each virus independently, using the infectivity at 5 or 6 h. However, if there was a differential effect of cyclosporin A on N74D virus over time, this effect could bias the normalized uncoating kinetics. Therefore, we examined the effect of CsA on wildtype and N74D infectivity in the parent CHME3 cell line over time by performing a CsA washout assay. Treatment with CsA decreased both wildtype and N74D infectivity at all time points examined compared to the ethanol control (Fig. 5). The magnitude of this decrease was determined by calculating ratio of infectivity for each time point examined (Table 2). In general, the CsA containing reactions exhibited 63–75% of the infected cells in the corresponding ethanol control (Table 2). This decrease was consistent across all time points examined and within a range of 70–75% during the first 2 h after infection when the majority of uncoating is observed (Fig. 4, Table 2).

**Table 2:** Ratio for the effect of CsA on HIV infectivity

Time (hr)	CsA/EtOH ratio	
	HIV-GFP	N74D
0	0.72	0.76
0.25	0.63	0.65
0.5	0.68	0.70
0.75	0.70	0.69
1	0.68	0.68
2	0.64	0.68
3	0.70	0.65
4	0.69	0.75
5	0.72	0.73

The magnitude of the effect of CsA was determined by calculating a ratio of infectivity (%GPP positive cells with CsA treatment/%GPP positive cells in EtOH carrier control) for each time point examined for HIV-GFP and N74D virus. In general, the CsA containing reactions exhibited 63–75% of the infected cells in the corresponding ethanol control

## Discussion

Here we have characterized HIV uncoating in the microglial cell lines CHME3 and C20. Similar to previous studies in owl monkey kidney (OMK) cells and HeLa cells, the half-life of uncoating was within an hour post infection and the majority of virus (~ 80%) uncoated within the first 2 h of infection (Fig. 1 and Table 1 [8, 40]). When comparing the kinetics, uncoating in CHME3 cells had average half-life of 52.46 min which is intermediate between what was previously observed in HeLa cells (36.8 min) and OMK cells (64 min [40]). The average half-life of uncoating in C20 cells was 34.95 min which is similar to the half-life in HeLa cells [40]. As uncoating follows viral fusion, alterations in the rate of viral fusion in different cell lines could alter the perceived rate of uncoating. We have previously observed this effect when characterizing the rate of uncoating of VSV-g pseudotyped virus and virus with wildtype HIV envelope in OMK cells, and the kinetics of uncoating in OMK and HeLa cells [8, 40]. Once the half-life of viral fusion was subtracted, the uncoating half-life in both CHME3 and C20 cells was 8.6 min (Table 1). These results suggest that in these microglial cell lines the uncoating process is initiated fairly early after viral fusion, within 8 min. These kinetics are most similar to HeLa cells where the difference in the average half-lives of viral fusion and uncoating was 8 min, while in OMK cells this difference was 45 min [40]. HeLa, CHME3, and C20 cells are all human cell lines, therefore it is likely they would display more similarities in uncoating compared to OMK cells.

We do not believe that expression levels of TRIM-CypA protein could account for the differences in uncoating kinetics between CHME3, C20, and OMK cells. The CsA washout assay was conducted under conditions in which TRIM-CypA restriction was not saturated, meaning that in the absence of CsA viral infectivity was completely inhibited. TRIM proteins rapidly associate with incoming virus [47]. Under these non-saturating conditions a sufficient amount TRIM-CypA protein was present to bind to the viral capsid and inhibit infectivity. The presence of additional TRIM-CypA protein should not have an additive effect on restriction to alter the detection of coated viral complexes in the CsA washout assay. A more likely hypothesis is that differences in the kinetics of uncoating between the microglial cell lines and OMK cells are due to the presence or absence of cellular factors. Several cellular motor and trafficking proteins have been identified which affect HIV uncoating [13–19]. Differential expression or the differential ability of HIV capsid to interact with owl monkey cellular factors could account for the overall delayed uncoating kinetics in OMK cells compared to CHME3 and C20 cells.

The process of reverse transcription has been shown to have a complicated interplay with the process of uncoating. Similar to previous studies in OMK cells, inhibition of reverse transcription in CHME3 and C20 cells delayed the process of uncoating in the CsA washout assay (Fig. 2 [8]). This effect of reverse transcription on uncoating is consistent with multiple studies using a variety of uncoating assays [8–10, 15, 30, 38, 45]. In addition, there was a rapid increase in the percentage of uncoated virions in the first hour after the nevirapine treatment was removed, indicating that a large number of viral complexes initiated uncoating after reverse transcription was allowed to proceed (Fig. 2). This result supports the model where minus strand strong stop DNA initiates uncoating as this early reverse transcription product could readily be generated in the hour after nevirapine removal due to its short length [30, 45]. Our results also concur with a recent study in which the fate of the capsid uncoating assay was used to examine the effect of reverse transcription on uncoating in CHME3 cells [15]. While this was not a kinetic analysis, inhibition of reverse transcription resulted in viral cores with increased amounts of CA protein at 3 h post-infection, indicating a delay in uncoating [15].

We next examined the effect of CA mutations on the process of uncoating. The N74D, E45A, and A92E mutants were chosen because they significantly altered the rate of uncoating in OMK cells in our previous study [40]. These mutants also can affect the infection of nondividing cells, utilization of nuclear import pathways, integration site selection, and interaction with cellular proteins that facilitate HIV infection [13, 20–23, 48, 52, 53]. Given the similarities in the kinetics of uncoating and effect of reverse transcription on uncoating in CHME3 and C20 cells, we tested these mutants only in the CHME3-TC cell line. N74D and E45A significantly altered the rate of uncoating compared to the parallel HIV-GFP control in CHME3 cells (Fig. 3). The effect of each mutation on uncoating was likely more severe than the data indicate because the CsA washout assay is based on infectivity. An infected cell in this assay indicates that the virus has successfully uncoated and then established a provirus to express the GFP reporter. Therefore, the uncoating kinetics of these mutants reveal the extent to which changes in uncoating can be tolerated while still resulting in productive infection.

Given that changes in the rate of reverse transcription can affect uncoating we tested progression of reverse transcription in the CA mutants. We found that these mutations did not significantly change the rate of reverse transcription at early timepoints post-infection in CHME3 cells (Fig. 4 [40]). Therefore, the alterations in uncoating for E45A and N74D virus were not due to alterations in reverse transcription. This result is similar to what was previously found in OMK cells for the E45A, N74D, and A92E mutations [40]. While some CA mutations have been shown to have altered capsid stability and changes in reverse transcription, the capsid mutations in this study provide a way to uncouple the process of uncoating from reverse transcription [7]. In previous experiments studying the effect of reverse transcription on uncoating in OMK cells, we found that each CA mutant virus had a similar delay in uncoating in response to nevirapine treatment [40]. Therefore, the effect of delaying uncoating in the CsA washout assay was dominant to the effect CA mutations. This result indicates that the contribution of reverse transcription to uncoating occurs before the effect of the N74D and E45A capsid mutations. Thus, these data also support the model by which the minus strand strong stop DNA initiates uncoating as this product is generated early in infection.

While the N74D mutation delayed uncoating in CHME3 cells, there are differences in the magnitude of this change compared to our previous studies [40]. In CHME3 cells N74D mutant virus uncoated with a half-life of 141 min compared to a half-life of 62 min for wildtype in the parallel assays (Fig. 3a). This ~ 2-fold change in uncoating kinetics is most similar to results in HeLa cells, while in OMK cells the N74D mutation only increased that half-life of uncoating by 50% [40]. This mutation has also been shown to delay uncoating in fluorescence microscopy based uncoating assays [30, 35]. The N74D mutation causes HIV to utilize a different nuclear import pathway than that mediated by the importin-β protein TNPO3, nucleocytoplasmic shuttle protein CPSF6, and nucleoporin NUP358 [20, 21, 23]. A study by Dharan et al. showed that knockdown of NUP358 and the microtubule kinesin-1 motor protein Kif5B delayed uncoating in HeLa cells using the fluorescence microscopy based in situ uncoating assay [13]. In this study the N74D mutation also prevented NUP358 and Kif5B from binding to the HIV capsid [13]. Therefore, a likely hypothesis is that the delayed uncoating kinetics of N74D mutant virus in CHME3 cells is due to the inability of this mutant to bind Kif5B or NUP358.

The E45A mutation produces a hyperstable capsid lattice with increased stiffness in atomic force microscopy assays [7, 68]. Like the N74D mutation, this mutation also results in the use of an alternate nuclear import pathway than that mediated by TNPO3, CPSF6, and NUP358 [20, 21]. In the CsA washout assay, the E45A mutation slowed the rate of uncoating in CHME3 cells with an increased half-life of 102 min compared to the wildtype control (Fig. 3b). This result is similar to our previous study, although there was a more modest delay in uncoating due to the E45A mutation in OMK cells [40]. E45A virus was also observed to uncoat slower than wildtype in fluorescence microscopy based uncoating assays [36, 38]. To determine whether capsid stability or disrupted interaction with cellular factors was responsible for the delayed uncoating of E45A mutant virus, we examined the uncoating kinetics of the E45A/R123T double mutant virus. R132T is a second site suppressor mutation that was isolated from serial passage of E45A mutant virus in culture [66]. In this study, the R132T compensatory mutation partially rescued infectivity and nuclear import defects of E45A mutant virus [66]. However, E45A/R132T virus still had a hyperstable capsid [66]. Surprisingly, we found that E45A/R132T virus uncoated with kinetics similar to wildtype (Fig. 3c). Therefore, the delayed uncoating kinetics observed in E45A virus were not due to a hyperstable capsid. This result suggests that overall capsid stability may not be a good predictor of uncoating kinetics, in agreement with other studies [24, 40, 66, 69]. The E45 and R132 residues are distant from each other but are both located near the NTD-NTD interface. As widespread structural changes are not observed in E45A or R132T mutant viruses, the altered chemical natures of these side chains were proposed to account for the effects of the mutations on replication [66]. The R132T mutation may restore an interaction surface needed for uncoating, and disruption of this region by the E45A mutation would result in delayed uncoating. The E45 residue has not been tested for NUP358 or Kif5B binding like the N74 residue, but mutation at this location may prevent the association with NUP358, Kif5B, or other members of the canonical nuclear import pathway that may be involved with uncoating. Supporting this idea, Yang et al. showed that the R132T mutation restored the ability of E45A virus to bind the drug PF74 [66]. PF74 directly binds to the conical capsid at a site where the cellular factors CPSF6 and the nucleoporin NUP153 bind to mediate nuclear import [25, 70, 71].

Yang et al. also found that E45A/R132T virus had less capsid disassembly compared to wildtype in an in vitro uncoating assay which may seem to contradict our results [66]. In this assay viral capsid cores were purified from virus, diluted in buffer, and the extent of disassembly after 30 min was assessed by detecting the remaining intact cores. However, this in vitro uncoating assay did not expose the capsid to cellular factors which may be needed for uncoating. In the absence of cellular factors E45A/R132T virus likely uncoated slower than wildtype and with a similar efficiency as E45A virus due to its increased capsid stability. In the CsA washout assay E45A/R132T virus uncoated at a rate that was not significantly different from wildtype because this assay is performed in cells which would allow interactions with cellular factors to impact uncoating (Fig. 3).

A92E displayed different behavior than what we have previously observed. In OMK cells, this mutation increased the rate of uncoating compared to wildtype [40]. However, in CHME3 cells this mutation did not significantly change uncoating kinetics (Fig. 3d). A92E has been implicated in infection of nondividing cells and altered use the cellular factor cyclophilin A for HIV infection [48, 52, 53]. Given the species difference between OMK and human cell lines, it is possible that A92E necessitates the use of different cellular factors, thus resulting in a differential effect of this mutation between cell lines.

Cyclophilin A (CypA) is an abundantly expressed cytoplasmic protein that binds the capsid to alter HIV replication. The exact role of CypA in viral replication is unclear as it may increase, decrease, or have no effect on infectivity depending on the cell line tested [21, 72–74]. In cells where cyclophilin A facilitates HIV infection it has been proposed to play a role in the processes of reverse transcription, uncoating, and nuclear import [21, 72–75]. CypA was able to modulate uncoating in a cell type dependent manner in the fate of the capsid assay and stabilize the capsid in an in vitro uncoating assay [74, 76]. Recently, CypA has been proposed to protect the capsid from restriction by human TRIM5α in primary human blood cells [77]. In the CsA washout assay, CsA is used to prevent binding of TRIM-CypA to the capsid which will also prevent cellular CypA from binding to the viral capsid. Therefore, observations cannot be made about the effect of CypA on uncoating of wildtype or CA mutant virus using the CsA washout assay. However, CsA treatment has been shown to decrease infectivity of N74D virus by ~ 2–3-fold in HeLa cells, whereas wildtype infectivity was slightly increased [67]. Because the uncoating of each virus is normalized independently, different sensitivities of wildtype and N74D virus to CsA could impact uncoating kinetics if this sensitivity changes over the time course of the assay. Therefore, we performed a CsA washout assay in the parent CHME3 cell line to determine the kinetics of CsA sensitivity in N74D and wildtype viruses. Compared to the carrier control ethanol, CsA treatment decreased the infectivity of both wildtype and N74D mutant virus in CHME3 cells (Fig. 4). This decrease was greater for wildtype virus compared to N74D mutant virus. However, these decreases in infectivity were consistently in the range of 63–75% over the time course of the CsA washout assay and 70–75% in the first 2 h of the assay for both viruses (Table 2). Therefore, in CHME3 cells sensitivity to CsA should not bias the uncoating kinetics for wildtype or N74D virus as determined by the CsA washout assay.

We chose to use the CsA washout assay to study uncoating as this assay is fairly high throughput compared to other uncoating assays, while allowing the assessment of uncoating kinetics at multiple timepoints post-infection [27]. In addition, the CsA washout assay provides a direct correlation between uncoating and successful infection of the cell. As the majority of HIV virions that enter cells do not establish productive infection, the characteristics of these defective virions could bias or obscure results. The CsA washout assay is indirect in that relies on the activity of TRIM-CypA to detect uncoating. TRIM-CypA self-associates into a hexagonal lattice that binds the hexameric CA lattice, but it is not known how much of the intact viral capsid must be present for TRIM-CypA lattice formation and restriction [63, 64]. In the CsA washout assay viral capsids may uncoat to varying extents in the cytoplasm before exposure to TRIM-CypA binding with the withdrawal of CsA. However, in a microscopy assay only intact capsids localized to the cytoplasmic bodies of the closely related TRIM family member rhesus TRIM5α, indicating that the majority of the capsid lattice may be required for TRIM protein binding [78]. Therefore, we propose that in the CsA washout assay TRIM-CypA binds intact capsids or viral complexes that have just started to uncoat. The CsA washout assay would then detect the initial destabilization of the capsid, an early step of uncoating. In support of this hypothesis, Mamede et al. observed uncoating kinetics similar to the CsA washout assay in live cell imaging assays [30]. In this study, uncoating was directly monitored by a green fluorescent protein fluid phase marker that localized to the interior of the conical capsid. Live cell fluorescence imaging tracked the loss of this marker at the initiation of uncoating and also determined which virions resulted in productive infection of the cell [30]. In addition, multiple studies using fluorescence microscopy and biochemical assays that directly detect the loss of CA have revealed a similar timing, effect of reverse transcription, effect of CA mutations, and effect of cellular factors on uncoating as in the CsA washout assay [8–10, 16, 38, 45, 48]. Therefore, while indirect the CsA washout assay provides a good monitor for the kinetics of successful uncoating in productively infected cells.

## Conclusions

In summary, we have characterized uncoating in the human microglial cell lines CHME3 and C20 which are natural targets of HIV infection. Similar to our previous results in cell lines that are not natural targets of HIV infection (OMK and HeLa cells), uncoating initiated within the first hour of infection and was facilitated by reverse transcription. These results support the model in which uncoating is initiated by early steps of reverse transcription in the cytoplasm and proceeds during transport of the viral complex to the nucleus. The CA mutations N74D and E45A delayed HIV uncoating, while the compensatory mutation R132T was able to rescue the uncoating kinetics of E45A mutant virus despite still having a hyperstable capsid. Discrepancies between the uncoating kinetics of CA mutant viruses in OMK and CHME3 cells reveals the importance of cellular factors in the process of uncoating. Specifically, the E45A/R132T mutant virus suggests that disrupted interactions with cellular factors, rather than capsid stability, is responsible for the delayed uncoating kinetics seen in E45A mutant virus. Future studies aimed at identifying these factors will be important for understanding the process of uncoating in cells, the influence of uncoating on other early steps of HIV replication, and the development of interventions to disrupt these processes.

## Data Availability

All data generated or analyzed during this study are included in this published article [and its supplementary information files].

## Abbreviations

CA: Capsid protein
CsA: Cyclosporin A
CypA: Cyclophilin A
HIV: Human immunodeficiency virus
NVP: Nevirapine
OMK: Owl monkey kidney
